# Chemotherapy in non-small cell lung cancer: opportunities for advancement

**DOI:** 10.1186/s40880-016-0119-x

**Published:** 2016-06-23

**Authors:** Mani Akhtari, Eric H. Bernicker, Bin S. Teh

**Affiliations:** Department of Radiation Oncology, Houston Methodist Hospital, Cancer Center and Research Institute, Weil Cornell Medical College, 6565 Fannin, Ste#DB1-077, Houston, TX 77030 USA; Department of Radiation Oncology, The University of Texas Medical Branch at Galveston, Houston, TX 77555 USA; Cancer Center, Thoracic Medical Oncology, Houston Methodist Hospital, Houston, TX 77030 USA

**Keywords:** Lung cancer, Chemotherapy, Radiotherapy

## Abstract

Locally advanced non-small cell lung cancer (NSCLC) continues to be a challenging disease to treat. With high rates of both local and distant failures, there is significant interest in finding more biologically active chemotherapy regimens that can contribute to reduce both failures. The phase III PROCLAIM trial, recently published in the *Journal of Clinical Oncology* entitled “PROCLAIM: randomized phase III trial of pemetrexed–cisplatin or etoposide–cisplatin plus thoracic radiation therapy followed by consolidation chemotherapy in locally advanced nonsquamous non-small-cell lung cancer”, compared two different chemotherapy regimens given concurrently with radiotherapy in patients with stage III non-squamous lung cancer: pemetrexed plus cisplatin versus cisplatin plus etoposide. Both groups received consolidation chemotherapy. After enrolling 598 of planned 600 patients, the study was stopped early due to futility as no difference was seen in the primary end-point of overall survival. Since PROCLAIM was designed as a superiority trial, these results suggest that pemetrexed regimens do not offer a clinical advantage over standard cisplatin plus etoposide. There are some subpopulations who might still benefit from pemetrexed, especially if clinicians are concerned about myelosuppression-related adverse events. Future trials are needed to investigate novel biologic agents and irradiation techniques that can result in more durable local and distant disease control in locally advanced NSCLC.

Lung cancer continues to be the leading cancer worldwide [1], with non-small cell lung cancer (NSCLC) representing approximately 85% of all lung cancers diagnosed in the United States [2]. However, overall outcomes in locally advanced NSCLC continue to lag behind improvements achieved in other cancer sites as indicated by the 5-year survival rate of patients with stage IIIA and IIIB NSCLC ranging from 2% [3] to 18% [4]. Regardless, the standard of care for locally advanced NSCLC remains concurrent radiotherapy, and yet the exact details of the concurrent chemotherapy continue to be the subject of much debate [5].

The question of sequential vs. concurrent chemotherapy has already been addressed in multiple randomized trials and meta-analyses [5–7]. Induction chemotherapy prior to concurrent chemoradiation has not shown significant benefit and is not considered standard in the algorithm of definitive treatment [8, 9]. Platinum-based doublets have been used in the majority of modern trials, including cisplatin plus etoposide [10] or weekly carboplatin plus paclitaxel [8]. Other attempts at modification of chemotherapy regimen, including consolidation chemotherapy following concurrent chemoradiation, have not shown a meaningful benefit either [11, 12]. Despite many advances, the rates of distant and local disease progression remain high, and efforts to find new areas for improvement have been numerous.

One of these efforts includes the use of pemetrexed, which is a potent anti-folate agent [13] and is currently approved for first-line treatment of metastatic non-squamous lung carcinoma and unresectable mesothelioma [14]. It has also shown efficacy in comparison to other regimens in the setting of advanced or metastatic non-squamous NSCLC [15]. The favorable toxicity profile of pemetrexed with concurrent irradiation, as well as its greater activity in metastatic non-squamous NSCLC, has raised the question of its possible role in definitive treatment of locally advanced NSCLC.

The PROCLAIM trial, recently published in the *Journal of Clinical Oncology* entitled “PROCLAIM: randomized phase III trial of pemetrexed-cisplatin or etoposide-cisplatin plus thoracic radiation therapy followed by consolidation chemotherapy in locally advanced nonsquamous non-small-cell lung cancer”, aimed to answer some of the above questions [16]. The trial enrolled patients with unresectable, stage III, non-squamous NSCLC who had an Eastern Cooperative Oncology Group performance status (PS) of 0 or 1. Some of the exclusion criteria included previous systemic chemotherapy or inability to meet dosimetric irradiation criteria, specifically if the volume of whole lung receiving 20 Gy or more exceeded 35%. Patients were randomly assigned on a 1:1 basis to either treatment group with the stratification criteria including PS, stage (IIIA vs. IIIB), and baseline positron emission tomography (PET) scan. The pemetrexed group received pemetrexed and cisplatin along with concurrent thoracic radiotherapy followed by four cycles of consolidation pemetrexed therapy. The etoposide group received cisplatin plus etoposide concurrently with radiotherapy followed by consolidation chemotherapy consisting of two cycles of a platinum-based doublet regimen. Thoracic radiotherapy consisted of 60–66 Gy, depending on meeting specific dose criteria, in 30–33 fractions. The primary end-point was designated as the overall survival (OS), with the assumptions of a superiority trial. A total of 600 patients were planned for enrollment, while assuming a minimum of 355 deaths and an 80% power to detect a hazard ratio (HR) of 0.74.

Overall, 598 patients were enrolled in the PROCLAIM trial with a median follow-up of approximately 23 months for both regimens. In the pemetrexed group, 80.9% of patients were able to complete treatment as opposed to 74.3% in the etoposide group. Both groups received similar dosages of thoracic radiotherapy in a similar pattern. Both regimens were well tolerated, but the pemetrexed group showed lower rates of neutropenia and febrile neutropenia than the etoposide group (18.4% vs. 28.7%, *P* = 0.005). Interestingly, the rate of grade 2 pneumonitis was significantly higher in the pemetrexed group than the etoposide group (11.0% vs. 5.5%). The primary end-point OS was not different between the two groups after 173 deaths (HR = 0.98, 95% confidence interval = 0.79–1.20, *P* = 0.831). The study was therefore stopped after the interim analysis after 798 patients were enrolled for futility of superiority of the pemetrexed group compared with the etoposide group.

Given the results of this trial, it appears that cisplatin plus etoposide remains the standard of care for non-squamous NSCLC. Although the pemetrexed group showed an overall better hematological toxicity profile compared with the etoposide regimen, it might be tempting to argue that pemetrexed plus cisplatin could be used in non-squamous NSCLC for concurrent treatment. However, it is important to keep in mind that even after nearly half of anticipated deaths, there was not even a trend for improvement in OS with pemetrexed. It is difficult to imagine that a HR of 0.74 could have been reached, even with a larger number of events.

There were some issues with the study that might have affected the overall outcomes in both groups. Initial positron emission tomography (PET) scan was not required of all patients. Furthermore, 4-dimensional computed tomography (CT) simulation and intensity-modulated radiation therapy, which are now frequently used as cornerstones of thoracic radiotherapy, were allowed but not required in the study. The consolidation regimens also varied widely in the etoposide group and divided between 3 different regimens, increasing the heterogeneity in that group. It is conceivable that the addition of different chemotherapeutic agents in the etoposide group raised the response rates and helped mask the superiority of pemetrexed, as essentially the trial was measuring continuation versus switch consolidation. In addition, approximately a quarter of the patients in the etoposide group eventually received pemetrexed when they progressed. Lastly, the anticipated number of deaths and HR was most likely set too high during the study design, minimizing any potential effect in a smaller population.

The PROCLAIM trial, while not demonstrating the superiority of pemetrexed plus cisplatin over cisplatin plus etoposide given concurrently with radiotherapy, established both regimens as reasonable alternatives for oncologists to consider. The pemetrexed regimen causes less hematological toxicity and does not cause alopecia, as well as requiring fewer visits to the infusion center, which could provide the oncologist with practical reasons choosing this regimen.

Many questions still remain in the treatment of locally advanced NSCLC. The rates of local relapse (37.3% in the pemetrexed group vs. 45.8% in the etoposide group) and distant metastases (31% in the pemetrexed group vs. 37% in the etoposide group) seen in the PROCLAIM trial point towards a better need for both local and distant disease control if meaningful gains are to be made in the treatment of locally advanced NSCLC. There are multiple ongoing studies investigating new strategies for better local control including using metformin as a radiosensitizer (NRG-LU001), photon vs. proton thoracic radiotherapy (RTOG 1308), and individualized adoptive radiotherapy by using a during-treatment PET (RTOG 1106). Immunotherapy has also started to play a promising role in the treatment of advanced lung cancer, and recent data have shown that it can contribute to improvements in outcomes. In the phase III START trial, anti-Mucin 1 agent tecemotide showed an improvement in OS compared with placebo in patients who had completed concurrent chemoradiation [17].

To make significant inroads in the treatment of locally advanced NSCLC, novel treatment strategies are needed given the minimal gains made recently in terms of prolonging OS. Now that ample evidence exists regarding the correct sequence of chemotherapy in locally advanced NSCLC and the most effective agents, future trials should direct their focus towards finding novel biologic agents and irradiation strategies that can contribute to both local and distant control.
